# The association between smart phone usage and body image distortion among adolescents and the effect of Social media

**DOI:** 10.1192/j.eurpsy.2023.299

**Published:** 2023-07-19

**Authors:** Y. J. Nam

**Affiliations:** psychiatry, Wonju Severance Christian Hospital; psychiatry, Yonsei University, Wonju College of Medicine, Wonju, Korea, Republic Of

## Abstract

**Introduction:**

What adolescents watch affects their body image. Smartphone is one of the most used tool in them today, allowing users to interact each other and share not only pictures but also sights.

**Objectives:**

The aim of this study is to evaluate the relationship between time/purpose (i.e. social media or the others) of smartphone usage and body image distortion revealed by weight control behavior. Differences in the effect of smartphone usage according to gender was also evaluated.

**Methods:**

The cross-sectional study obtained data from the 2017 Korea Youth Risk Behavior Web-based Survey, a nationally representative survey targeting middle and high school students (N=62,276). We categorized the time using a smartphone into 4 groups: less than 1 h, 1-2 h, 3-4 h, and 5 h or more a day. The students were divided into two groups according to whether their main purpose of using a smartphone is social media or not. We defined that adolescents have body image distortion when they attempted to lose weight in the last 30 days, even though they were not obese (BMI < 95th percentile). We classified inappropriate weight control behavior as using: fasting for at least 24 hours, taking over-the-counter diet pills, taking laxatives or diuretics, vomiting, and eating only one food. We performed logistic regression analysis to evaluate the association between time using a smartphone and body image distortion expressed as all kinds of weight control behavior, adjusting for age, gender, residential area, family economic status, hours of sleep, current alcohol consumption, smoking and day time activity. Furthermore, we repeated logistic regression analysis to evaluate the association between time using a smartphone and inappropriate weight control behavior. Analyses were also stratified according to gender and main purpose of using a smart phone.

**Results:**

Smartphone usage time was significantly associated with body image distortion and inappropriate weight control behavior in total group and female adolescents (P <0.05). The association between inappropriate weight control behavior and smartphone usage time is significant even in male adolescent if they use a smartphone more than 5 hours a day. The association between inappropriate weight control and smartphone usage time is also significant when they are not obese. The odd ratio of inappropriate weight control behavior in not obesity group was higher when social media is the main purpose of using a smartphone than the others (OR 1.390 in total group, 1.491 in girls, P <0.001).

**Conclusions:**

Using a smartphone and social media too much is associated with body image distortion and inappropriate weight control behavior in adolescents. It is necessary to reduce excessive smartphone use and educate media literacy for healthy body awareness.

**Disclosure of Interest:**

None Declared

